# Supplementary material for: A Global Screen for Assembly State Changes of the Mitotic Proteome by SEC-SWATH-MS
Source: Cell Syst. 2020 Feb 26;10(2):133–155.e6. doi: 10.1016/j.cels.2020.01.001 (PMC7042714; doi:10.1016/j.cels.2020.01.001)

Q9P013 | CWC15_HUMAN | CWC15 C11orf5 AD−002 HSPC148
Monomer MW [kDa]: 26.624 Monomer expected elution fraction: 52

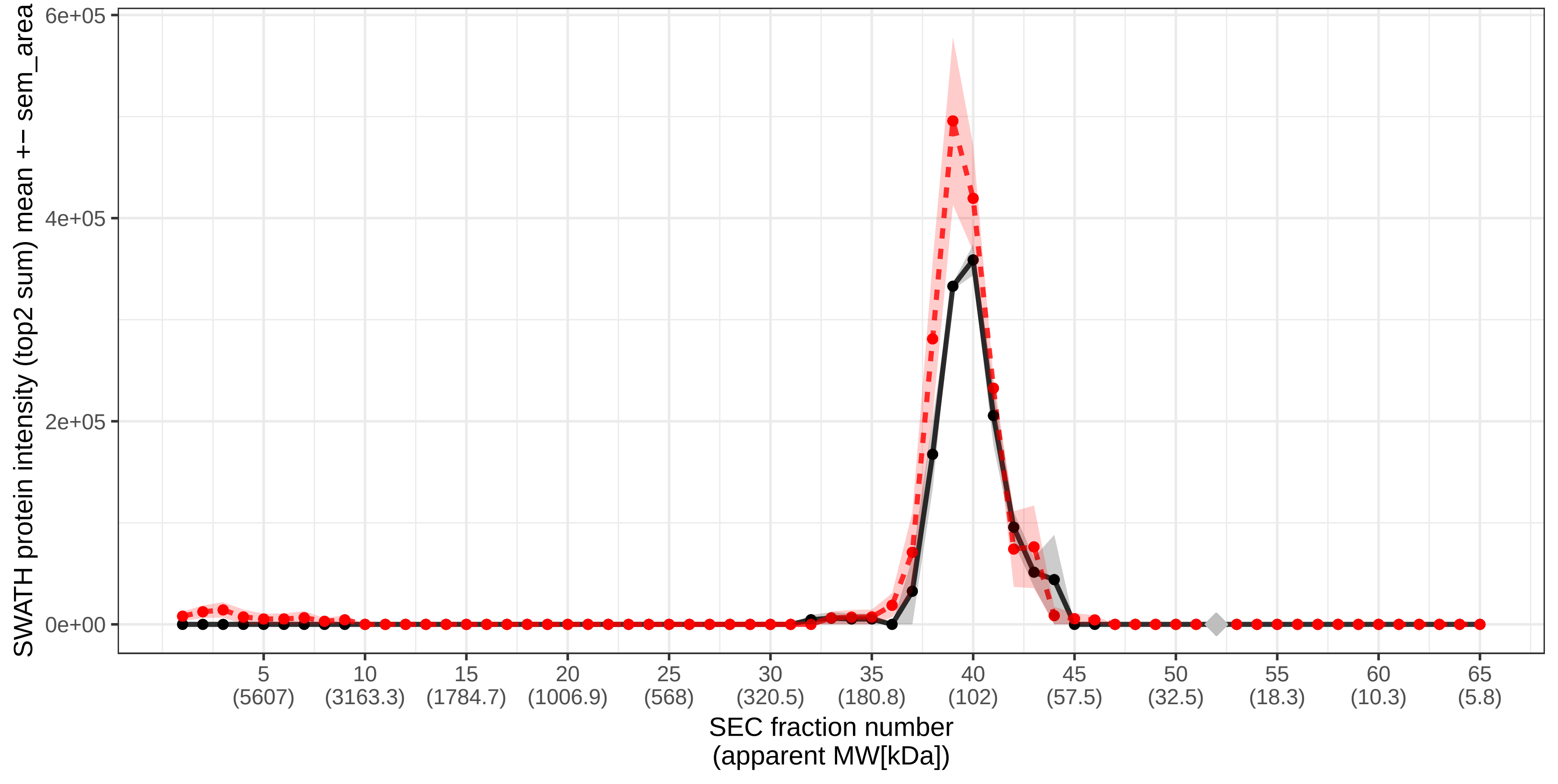

Supplement: Data S1. SEC-SWATH-MS Protein Chromatograms, Related to Figure 1 [file mmc6.zip › SECchrom_Q9P013_CWC15_HUMAN_CWC15_C11orf5_AD-002_HSPC148.pdf]
